# Cathepsins in cellular entry of human pathogenic viruses

**DOI:** 10.1128/jvi.01642-24

**Published:** 2025-03-26

**Authors:** Tejal Pathak, Sampurna Pal, Indranil Banerjee

**Affiliations:** 1 Cellular Virology Laboratory, Department of Biological Sciences, Indian Institute of Science Education and Research (IISER) 124268, Mohali, Punjab, India; Indiana University Bloomington, Bloomington, Indiana, USA

**Keywords:** cathepsin, virus, viral entry, infection, inhibitor

## Abstract

In the life cycle of a virus, host cell entry represents the first step that a virus needs to undertake to gain access to the cell interior for replication. Once a virus attaches itself to its target cell receptor, it activates endogenous cellular responses and exploits host cell factors for its internalization, fusion, and genome release. Among the host factors that critically contribute to the viral entry processes are cathepsins, which are the most abundant endo/lysosomal proteases with diverse physiological functions. This review summarizes previous findings on how different cathepsins contribute to the host cell entry of human pathogenic viruses, focusing on their specific roles in the entry processes of both enveloped and non-enveloped RNA viruses. A comprehensive knowledge of the functions of different cathepsins in viral entry will provide valuable insights into the molecular mechanisms underlying viral infections and can be useful in the development of new antiviral strategies.

## INTRODUCTION

To infect a target cell, a virus particle will first need to enter the cell, breach membrane barriers, and transmit its genome and accessory proteins to the cell. Upon successful transmission of genome and accessory proteins to the target cells, viruses replicate to produce progeny virions, which are subsequently released into the extracellular environment to initiate new infection in the noninfected cells. Being simple in structure and lacking metabolic and locomotory activities, viruses alone are limited in their capacity to undertake the processes necessary to complete their life cycle. Therefore, viruses, as passive entities, evolved elegant strategies to exploit the machinery and behavior of the host cells to gain entry and execute their infection program. Upon recognizing a permissive cell, a virus activates endogenous cellular responses that facilitate viral entry and help the virus to breach membrane barriers and deliver its genome into the cytosol or the nucleus. Although entry does not guarantee successful establishment of infection (a virus needs to evade antiviral and immune responses inside the cell before it can produce its progenies), it is the first step in the viral life cycle that a virus needs to undertake to gain access to the cell interior.

During host cell entry, while some viruses directly fuse at the plasma membrane, others utilize host endocytic pathways for their internalization and subsequent fusion at the endosomal membrane. Fusing either at the plasma membrane or endosomal membrane, viral capsids undergo uncoating, liberating their genome into the cytosol or, in some cases, into the nucleus (1, 2). Since viral entry is dependent on a limited number of cellular entry pathways and since multiple viruses co-opt common entry routes, targeting viral entry has several prophylactic and/or therapeutic implications. First, a block in viral entry should inhibit the downstream infection processes, limiting virus replication and spread. Second, targeting one or a combination of pathways that viruses utilize for internalization, it should be possible to broadly restrict a wide range of viruses (2).

Outside the host cell, the viral genome inside the capsid remains in an inactive and condensed form, protected from different environmental stresses (3). During the entry process, many molecular interactions that were established during the assembly of the virion must be reversed. Undoing the stabilizing interactions in the virus particle is important to liberate its genome and accessory proteins into the target cell. To achieve this feat, viruses locked in a metastable state depend on appropriate cellular cues and host molecules that trigger major conformational changes in the viral proteins (VPs), enabling the viruses to overcome the cell membrane barriers, destabilize the nucleocapsids, and transfer their genome into the cell (4). Viral entry and capsid uncoating follow tightly regulated consecutive steps, and each step is dependent on various cellular cues including receptor cues (plasma membrane-associated molecules), chemical cues (endosomal pH and other ions), facilitators (host proteins, lipids, and sugars), and enzymatic cues (host enzymes including proteases) (5). Among several proteases that either directly act on viruses or regulate key cellular processes to promote viral entry and penetration are endo/lysosomal cathepsins.

In this review, we present a compendium of previous findings that highlight the key functions of cathepsins in the entry of human pathogenic RNA viruses. Beginning with an overview of the general roles of cellular non-cathepsin proteases in viral entry, we specifically focus on how cathepsins, the endo/lysosomal proteases, contribute to the sequential steps of viral entry: attachment, internalization, fusion, uncoating, and genome release. We describe the general functions of cathepsins, their biosynthesis, and trafficking to different cellular compartments and highlight the mechanisms by which cathepsins facilitate the entry of both enveloped and non-enveloped RNA viruses. This review also discusses how the activity of specific cathepsins contributes to structural changes in viruses, enabling their transition from a stable, extracellular form to an entry-competent configuration, critical for breaching membrane barriers, uncoating, and genome release.

## VIRUS PROCESSING BY NON-CATHEPSIN PROTEASES

Before a virus can utilize the host cell’s resources and rewire them for its replicative program, it needs to overcome the host membrane barriers for genome release. Based on the presence or absence of lipid envelope, viruses have evolved distinct strategies for their initial attachment to host receptors and for their subsequent internalization and penetration. Enveloped viruses are equipped with membrane-anchored viral proteins that interact with the cell surface receptors, whereas the nonenveloped viruses bind to the receptors via their capsid surface or projections of the capsids (3, 4). However, many viruses cannot achieve successful entry unless their components are “primed” prior to the initiation of the entry process. Processing of the viral surface proteins, essential for viral entry, is dependent on multiple enzyme classes including the proteolytic enzymes called proteases that act by cleaving peptide bonds in proteins. Following synthesis, many viral surface proteins remain in an inactive form. The inactive surface proteins are cleaved by host cell proteases in the infected cells and are activated before virus assembly. In the absence of specific proteases, viral surface proteins will fail to be processed, and the unprocessed proteins will be incorporated into the assembling viruses. When the newly egressed viruses with inactive surface proteins encounter their target cells, their surface proteins must be activated by host proteases for successful entry.

Host cell proteases include the endo/lysosomal cathepsins. However, in this section, we provide a few examples of how non-cathepsin proteases mediate cellular entry of some enveloped and non-enveloped viruses. Enveloped viruses have glycoproteins (GPs) anchored in their lipid envelope that interact with the cell surface receptors for binding. The host cell entry of severe acute respiratory syndrome coronavirus-2 (SARS-CoV-2) initiates with the engagement of the viral spike (S) protein with the angiotensin-converting enzyme 2 (ACE2) receptor present on the host cell surface. SARS-CoV-2 can either directly fuse at the plasma membrane (6) or enter the cell by clathrin-mediated endocytosis (CME) (7) or clathrin-independent carrier/GPI-anchored protein-enriched early endocytic compartment (CLIC/GEEC) pathway (8). The viral spike glycoprotein S undergoes two cleavage events to enable successful entry of coronavirus into a cell. The transmembrane protease serine 2 (TMPRSS2) present at the plasma membrane cleaves the spike protein at the S2 cleavage site. This cleavage exposes the fusion peptide of the spike S2 domain, which in turn triggers viral fusion at the plasma membrane (6). In the absence of TMPRSS2 at the cell surface, the S protein does not undergo proteolytic processing upon binding to ACE2, and therefore, the virus cannot fuse at the plasma membrane. SARS-CoV-2 with the unprocessed S protein typically enters the host cells via endocytic routes, and upon sorting into endosomes, it becomes fusion-competent by the proteolytic activity of the endosomal cathepsins acting on the S protein (9, 10). Upon fusion of the viral and host plasma/endosomal membranes, the viral RNA is released into the cytoplasm for replication. Once the virus replicates and produces its progeny virions, the polybasic site at the S1-S2 boundary of the S protein of the progeny virion is cleaved by the host cell protease furin in the endoplasmic reticulum (ER)-Golgi intermediate compartment (ERGIC) and the Golgi complex. This cleavage event primes the S protein of the progeny virions, enabling them to infect uninfected cells upon their release from the producer cell (6).

The surface glycoprotein hemagglutinin (HA) of influenza A virus (IAV) is responsible for binding to sialylated receptors of the host cell. This protein also mediates fusion of the viral envelope with the endosomal limiting membrane upon exposure to low pH in the acidic lumen of the late endosome (11). Although IAV majorly uses CME to enter its host cell (12), it also uses macropinocytosis (13) or other non-clathrin-dependent and non-caveola-dependent endocytic pathways (14) as alternative entry routes. After reaching the late endosome, the acidic pH in the endosomal lumen triggers conformational rearrangements of the trimeric HA molecules. The low pH-induced conformation of HA exposes the viral fusion peptide, which catalyzes fusion between the viral envelope and endosomal membrane. Upon fusion at the late endosome, IAV capsid undergoes disassembly, and the viral ribonucleoproteins (vRNPs) are released into the cytosol. The liberated vRNPs are subsequently trafficked to the nucleus for viral replication and transcription (15). During the late phase of infection, HA is synthesized in the ER as a fusion-incompetent precursor termed HA0. In order to become fusion-competent, HA0 must undergo catalytic cleavage into HA1 and HA2 subunits. As HA traffics from the ER to the *trans*-Golgi network (TGN), it is catalytically processed in the TGN *en route* to the plasma membrane by various proteases including furin, TMPRSS2, and possibly TMPRSS4. Additionally, HA can also be cleaved by the human airway trypsin-like protease present at the plasma membrane (11).

Unlike the enveloped viruses, the non-enveloped viruses lack host-derived lipid envelope and are therefore unable to utilize membrane fusion for penetration and genome delivery. Although lacking a lipid envelope, non-enveloped viruses are equipped with specialized “penetration proteins” on their surface, which are critical for breaching membrane barriers and viral genome delivery (16). The non-enveloped reovirus (ReV) initiates infection by attaching to the cellular tight junction protein JAM-A and cell surface carbohydrates via its filamentous coat protein, σ1. Upon attachment to the cell surface, ReV is internalized by β1 integrins via CME. After trafficking to the acidic endocytic compartment, the endosomal cysteine proteases remove the viral outer capsid protein σ3. Removal of σ3 exposes the viral membrane penetration protein μ1, which is then proteolytically processed, leading to the generation of infectious subvirion particle (ISVP). Penetration of the endosomal membrane by the processed outer capsid protein μ1 enables the release of the viral core into the cytoplasm (17). Another example of proteolytic processing of viral protein of a non-enveloped virus is the cleavage of the VP4 spike protein of rotavirus (RV). During RV entry, the host protease trypsin cleaves VP4 into the subunits VP8 and VP5. Upon cleavage, the VP8 domain of VP4 mediates initial attachment of the virus to the target cell surface, and the VP5 domain of VP4 and VP7 subsequently interacts with the downstream co-receptors to facilitate viral internalization (18).

In the above examples, we highlighted the functions of some key cellular non-cathepsin proteases in the activation of surface proteins of some viruses. Although several host proteases have been implicated in processing surface proteins of a multitude of viruses during entry, this review specifically focuses on the functions of the cathepsin family of proteases in viral entry.

## CATHEPSINS: AN INTRODUCTION

**Fig 1:**
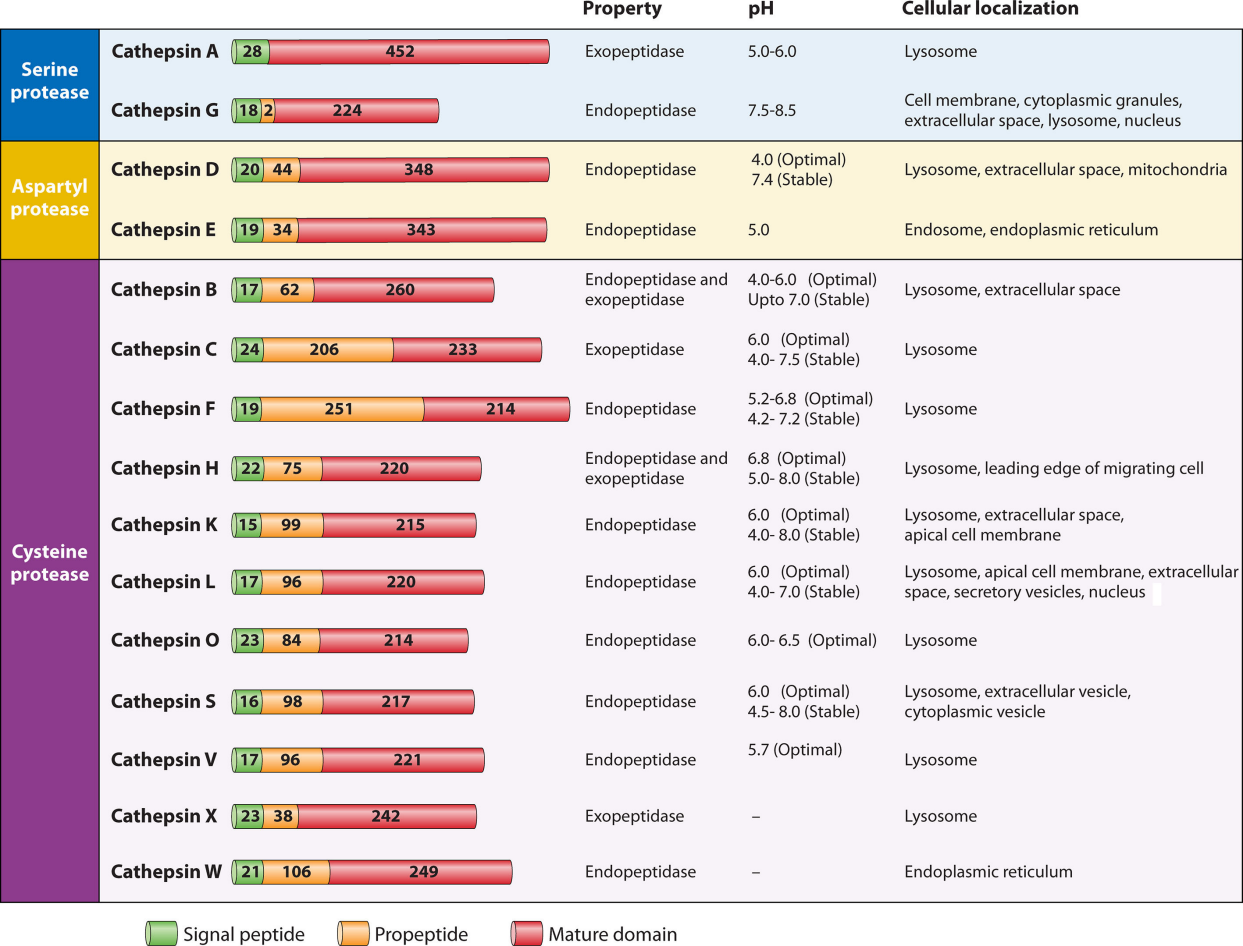
Classification, domain organization, biochemical properties, and subcellular localizations of human cathepsins. Structurally, cathepsins are classified into three classes: serine, aspartic, and cysteine cathepsins. Cathepsins are synthesized as a polypeptide chain consisting of a signal sequence (green), a propeptide (orange), and a mature catalytic domain (red). The amino acid length of each domain is indicated. The biochemical properties (endo or exopeptidase function), the activity pH, and the cellular localizations of the corresponding cathepsins are summarized.

Cathepsins are the most abundant and heterogeneous group of proteases present in acidic endosomes/lysosomes with astonishingly broad range of functions, including, but not limited to, degradation of superfluous and misfolded proteins, protein turnover, energy metabolism, immune responses, and viral infection regulation (19, 20). Although cathepsins are majorly found in an active form in the acidic lumen of lysosome, some cathepsins can also exhibit catalytic activity outside lysosome, and under certain circumstances, some are secreted outside the cell (21–24). More than 20 cathepsins have been identified to date in plants and animals, but the repertoire of human cathepsins comprises 15 members, which are structurally classified into three distinct groups based on the amino acids present in their active sites. The classification, structural schematic representation, activity pH, and subcellular localization of the human cathepsins are shown in Fig. 1.

Serine proteases (cathepsins A and G) are the most abundant cathepsins (31% of the total proteases expressed in humans), followed by aspartyl proteases (cathepsins D and E) that make up to 4% of total proteases. Constituting up to 25% of total proteases, cysteine proteases represent the most diverse group of cathepsins including 11 members: cathepsins B, C, F, H, K, L, O, S, V, X, and W (20, 25, 26). While the majority of the cathepsins are endopeptidases that act by cleaving peptide bonds within a polypeptide chain, cathepsins A, B, and X are carboxypeptidases that function by removing amino acids from the C-terminus, and cathepsin H is an aminopeptidase that acts by cleaving amino acids from the N-terminus (27, 28). Cathepsins B and H, in addition to showing carboxypeptidase and aminopeptidase activity, respectively, also exhibit endopeptidase activity (27). While most of the cathepsins exhibit optimal activity in the acidic environment of lysosomes (pH 4.5–5.0), some also show activity outside the lysosomal pH range. For example, cathepsin S exhibits optimal activity at pH 6.5, whereas cathepsin D is found to show activity even at pH 7.4, despite being optimally active at pH 4.0 (29–31). Cathepsin H shows optimal activity between pH 5.5 and 7.0 (32), and cathepsin K maintains catalytic activity even between pH 7.0 and 8.0 (33). The catalytic activity of cathepsins in the wide range of pH indicates that many cathepsins retain their functions even outside the endosomal or lysosomal compartments.

## CATHEPSIN BIOSYNTHESIS, MATURATION, AND SORTING

Almost all cathepsins are synthesized through a common pathway as precursor molecules called prepropeptides. The prepropeptide consists of an N-terminal hydrophobic signal sequence of 20–25 amino acids, followed by a precursor peptide and a catalytic domain. The signal sequence directs the entry of the zymogenic form of cathepsin into the lumen of the ER, where it is co-translationally cleaved by the activity of signal peptidase. Concurrently, oligosaccharides are added to procathepsins. The glycoprotein with processed glycan chains leaves the ER via vesicle-mediated transport and reaches the *cis* face of the Golgi complex by transiting through ERGIC. Inside the Golgi lumen, mannose glycans are phosphorylated, generating mannose-6-phosphate (M6P) (34). M6P is formed by the covalent addition of phosphate groups to select mannose residues by the sequential activity of two enzymes. First, GlcNac-1-phosphotransferase, encoded by the *GNPTAB* and *GNPTG* genes, mediates the transfer of GlcNac-1-phosphate from UDP-GlcNac to select mannoses at the C6 hydroxyl groups generating the phosphodiester forms. Second, the M6P uncovering enzyme *N*-acetylglucosamine-1-phosphodiester α-*N*-acetylglucosaminidase, present in the TGN and encoded by the *NAGPA* gene, catalyzes the hydrolysis of *N*-acetylglucosamine-1-phosphodiester, exposing the M6P residues on the high mannose type oligosaccharide (35, 36).

**Fig 2:**
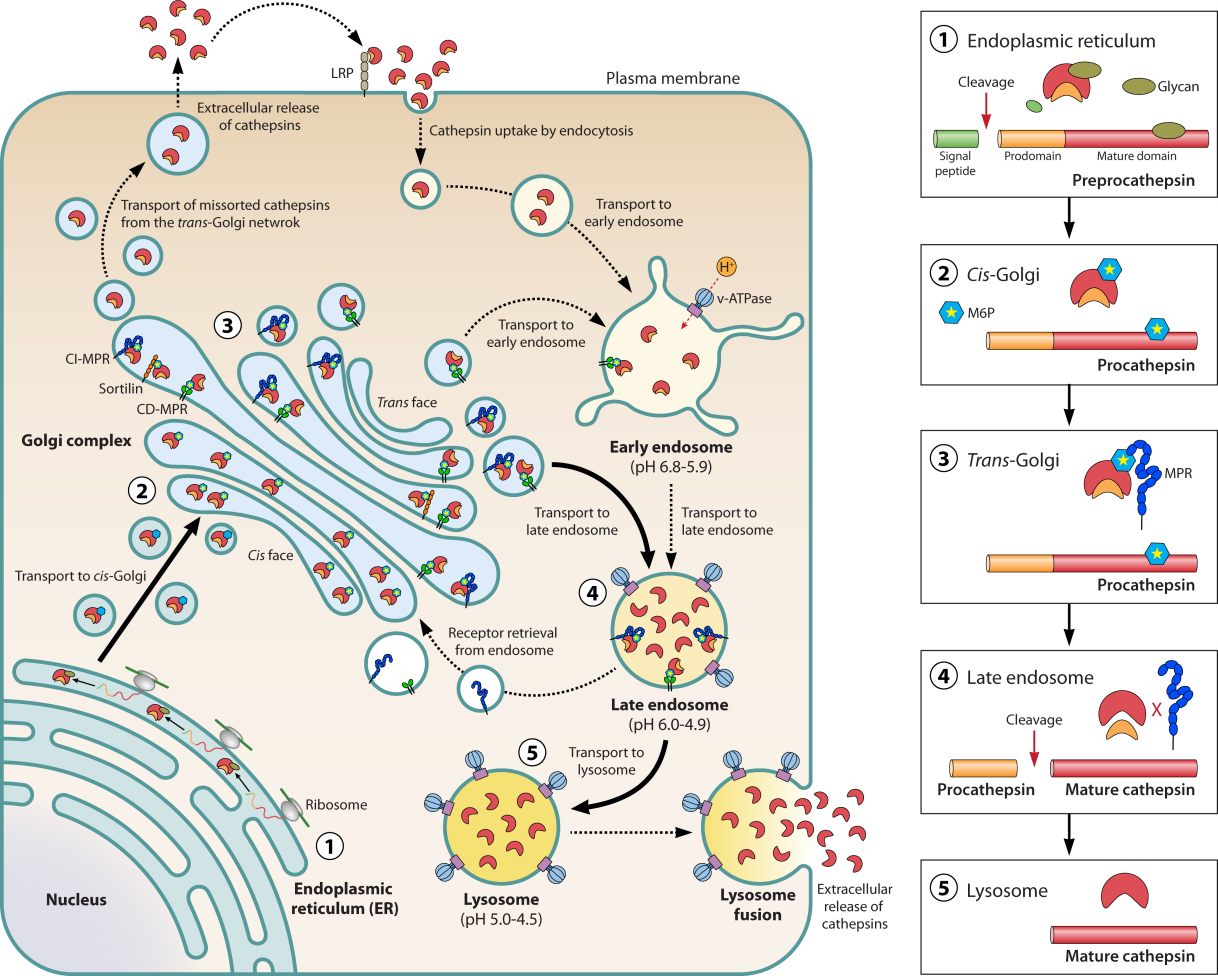
Schematic representation of cathepsin biosynthesis and transport pathways. Catalytically inactive preprocathepsins are synthesized in the lumen of the ER. (1) Upon synthesis, the N-terminal signal peptide (green in preprocathepsin) is co-translationally cleaved in the ER lumen, generating procathepsins. Procathepsins are glycosylated (myrtle green) in the ER lumen and are transported to the Golgi complex via vesicle-mediated transport. (2) In the *cis*-Golgi, select mannose residues on the glycan chains conjugated to procathepsins are phosphorylated (yellow star in blue hexagon). (3) Phosphorylated procathepsins are recognized by the Golgi-resident M6P receptors including the CI-MPR, CD-MPR, sortilin, LRP-1 etc. (4) The receptor-bound procathepsins are transported from the TGN to the late endosomes. Upon arrival at the acidic late endosomes, the low pH of the endosomes causes the dissociation of the procathepsins from their sorting receptors. Within the acidic compartments, the propeptide (orange) is proteolytically removed, rendering the cathepsins mature and proteolytically active. (5) Active cathepsins are transported to the lysosomes. The unbound sorting receptors are retrieved from the endosomes and are recycled back to the TGN via retromer-coated vesicles. Active cathepsins can be secreted outside the cell via lysosomal exocytosis. Alternatively, procathepsins can be mis-sorted from the TGN and are released into the extracellular space via secretory vesicles. The secreted procathepsins and active cathepsins are recaptured at the plasma membrane by CI-MPR or LDL receptor-related proteins (LRPs) and are endocytosed and retrogradely transported to the endo/lysosomes.

The exposed M6P residues on procathepsins are subsequently recognized by M6P-specific receptors (MPRs), which are found in the *trans*-Golgi compartments, plasma membrane, endosomes, but not in the lysosomes. Two distinct MPRs, a 46 kDa cation-dependent MPR (CD-MPR) and a 300 kDa cation-independent MPR (CI-MPR), recognize the M6P residues on procathepsins and mediate their transport from the TGN to endosomes. MPR-bound procathepsins are sorted by the adaptor protein complex-1 into clathrin-coated intermediates, which exit the TGN and deliver the cargoes to the endosomal compartments. However, a small fraction (5%–20%) of newly synthesized lysosomal enzymes escape the recognition by MPR and are secreted out of the cell (37). The MPR-bound procathepsins either are directly delivered to endosomes or reach the plasma membrane from which they are internalized via clathrin-coated vesicles, which deliver them to endosomes. The low pH in the endo/lysosomal compartments causes detachment of the ligands from the MPRs, and the MPRs are retrieved and recycled back to the TGN to engage in further rounds of cathepsin transport (36). However, incomplete sorting of MPRs at the TGN or a failure in their recycling from endosomes may cause accumulation of a fraction of MPRs at the cell surface (38). While most of the cathepsins use MPR-dependent pathways, alternative receptors like sortilin, lysosomal integral membrane protein type-2 (LIMP-2), low-density lipoprotein receptor (LDLR), and low-density lipoprotein receptor-related protein 1 (LRP1) are also reported to transport specific cathepsins to endosomes (39–42). The biosynthetic and transport pathways of cathepsins are shown in Fig. 2.

Once cathepsins reach their designated endosomal compartments, the acidic environment inside the endosomal lumen induces cleavage of their N-terminal propeptide, resulting in the activation of the enzymes. This low pH-induced cleavage of the propeptide occurs by either auto-activation through the catalytic activity of the same enzyme or trans-activation, arbitrated by other molecules of the same cathepsin or different cathepsins. Cathepsins B, H, L, K, and S undergo auto-activation, while the activation of cathepsins C and X is dependent on the activity of cathepsins L and S, respectively (20). Interestingly, cathepsin D activation involves a combination of a partial auto-activation step, generating a pseudo cathepsin D and an enzyme-assisted complete maturation step. The partially auto-activated pseudo cathepsin D undergoes processing by cysteine and/or aspartic proteases, transits through an intermediate stage, and finally becomes mature and active (43).

**Fig 3:**
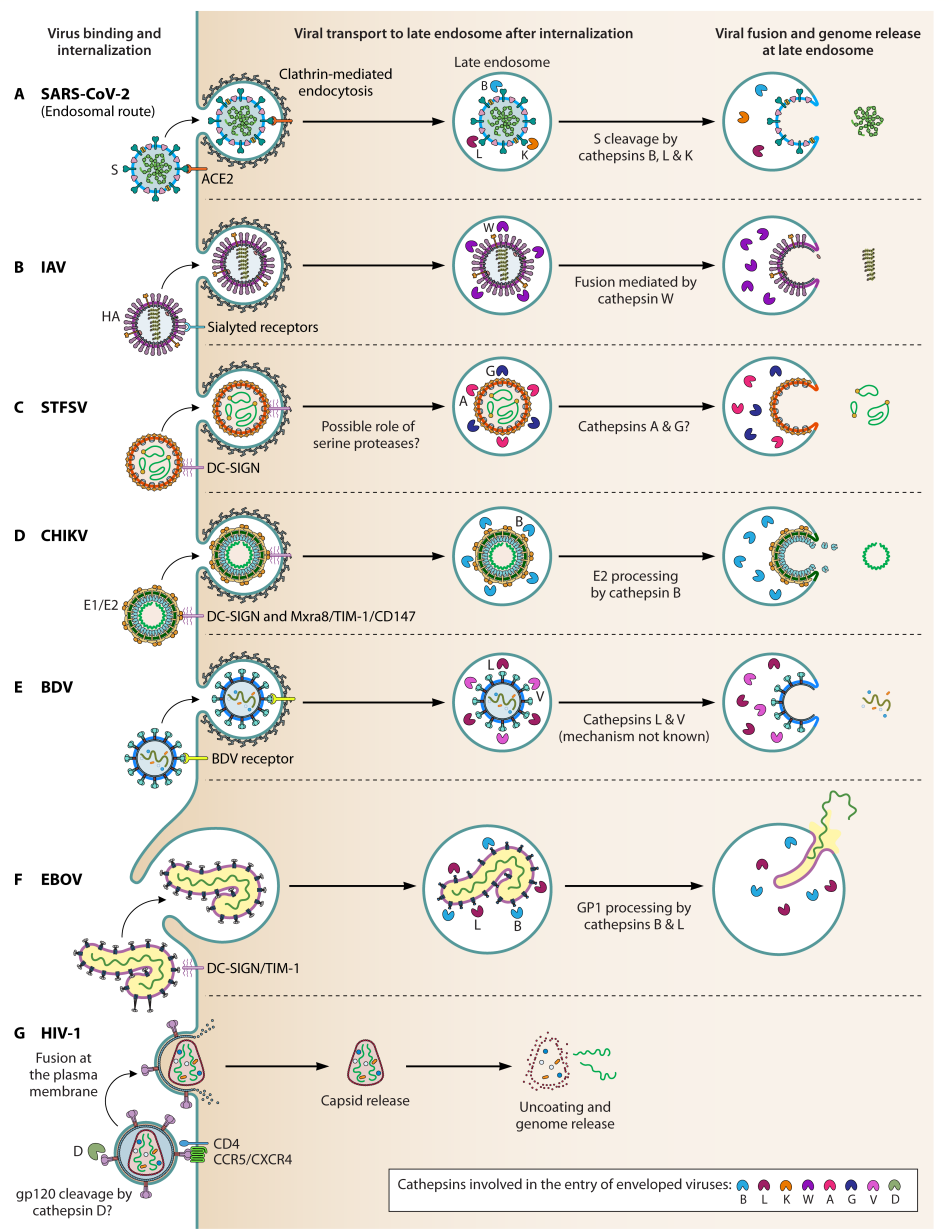
Schematic diagram showing the involvement of cathepsins in the cellular entry of enveloped RNA viruses. Upon attachment to the respective receptors on the plasma membrane, enveloped RNA viruses including SARS-CoV-2, IAV, severe fever with thrombocytopenia syndrome virus (SFTSV), Chikungunya virus (CHIKV), Borna disease virus (BDV), and Ebola virus (EBOV) are endocytosed by different endocytic pathways such as clathrin-mediated endocytosis or macropinocytosis, and are trafficked to the late endosomes at which they fuse and release their genome near the perinuclear region. Human immunodeficiency virus type 1 (HIV-1) majorly fuses at the plasma membrane and transmits its genome into the cytosol near the cell periphery. During intracellular trafficking, different cathepsins play distinct roles in processing the viral components, which facilitate viral fusion and uncoating. (A) Proteolytic cleavage of S protein in the endo/lysosome by cathepsins L, B, and K during SARS-CoV-2 entry. (B) Involvement of cathepsin W in the fusion of IAV at the late endosome. (C) Proteolytic activity of cathepsins A and G in the cytoplasmic release of the SFTSV genome. (D) Possible activity of cathepsin B in CHIKV uncoating. (E) Possible activity of cathepsins B and L in BDV genome release. (F) Cleavage of the EBOV glycoprotein by cathepsins B and L. (G) Extracellular cleavage of gp120 by cathepsin D during HIV-1 entry.

**Fig 4:**
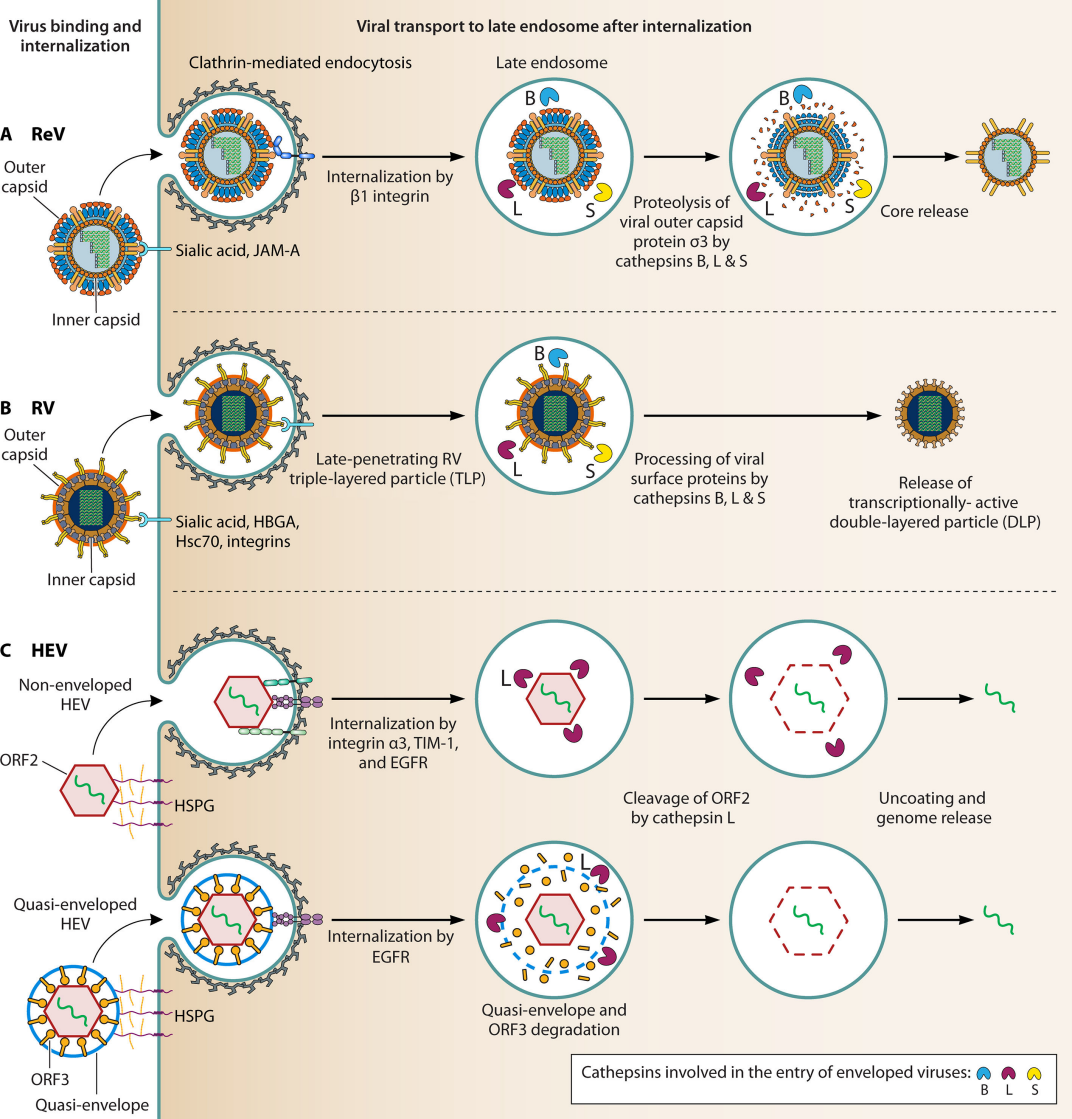
Schematic diagram showing the involvement of cathepsins in the cellular entry of non-enveloped RNA viruses. After binding to the cell surface by specific attachment factors, non-enveloped RNA viruses including ReV, RV and hepatitis E virus (HEV) are internalized via clathrin-mediated endocytosis. During intracellular trafficking, different cathepsins play distinct roles in processing the viral surface proteins or capsids, facilitating viral core/genome release into the cytosol. (A) Processing of ReV virion by cathepsins B, L, and S during entry. (B) Possible conversion of late-penetrating RV triple-layered particle (TLP) to a transcriptionally active, double-layered particle (DLP) by cathepsins B, L, and S. (C) Cathepsin L processes the HEV capsid protein ORF2, facilitating the dissociation of the viral RNA from the capsid during entry.

Accumulating evidence suggests that cathepsins present in the endo/lysosomal system or in unexpected locations such as cytosol or extracellular space provide critical assistance to the invading viruses to establish and develop fulminant infections. Although the earlier reviews broadly addressed the involvement of lysosomal proteases in viral infections, this review specifically focuses on the roles of cathepsins in cellular entry of human pathogenic RNA viruses, both enveloped and non-enveloped (schematically represented in Fig. 3 and 4).

## ROLE OF CATHEPSINS IN ENVELOPED RNA VIRUS ENTRY

### SARS-CoV-2

SARS-CoV-2 is a positive-sense, single-stranded RNA virus that belongs to the family *Coronaviridae*. To infect the target cells, SARS-CoV-2 relies on its obligate receptor ACE2, present on the host cell membrane (44, 45). The SARS-CoV-2 S protein interacts with the ACE2 receptor on the cell surface, following which, the virus either penetrates at the plasma membrane or is internalized through endocytic pathways. The key determinant of viral fusion at the plasma membrane is the presence or absence of the furin-like protease TMPRSS2 on the cell surface. In cells expressing TMPRSS2, SARS-CoV-2 typically fuses at the plasma membrane, but in the TMPRSS2-deficient cells, the virus preferentially internalizes through the endocytic routes (6). Also, the viral entry routes (fusion at the plasma membrane or uptake by endocytic pathways) can vary depending on the viral strains. Although the SARS-CoV-2 Omicron strain is reported to fuse at the plasma membrane, it preferentially uses the endosomal route for penetration (46, 47). SARS-CoV-2 that enters by endocytosis is critically dependent on endosomal cathepsins for processing and activation of its S protein.

Inside the endo/lysosomal compartment, cathepsins proteolytically cleave the S protein, exposing the fusion peptide on the S2 subunit. The activated fusion machinery of the virus then mediates fusion between the viral envelope and the endosomal limiting membrane, leading to viral genome release into the cytosol (6). Among the cellular cathepsins, cathepsin L was first identified as a critical mediator of SARS-CoV-2 endosomal fusion (48–50). Using SARS-CoV-2 pseudovirus, Ou et al. investigated the effect of a broad spectrum inhibitor of cathepsins L, H, B, and calpain (E64d), a cathepsin L inhibitor (SID 26681509), and a cathepsin B inhibitor (CA-074) in human embryonic kidney (HEK) cells stably expressing human ACE2 (HEK-hACE2). While broad inhibition of cathepsins by E64d or specific inhibition of cathepsin L by SID 26681509 markedly reduced SARS-CoV-2 pseudovirus infection, inhibition of cathepsin B by CA-074 did not show any significant reduction in infection, indicating that cathepsin L plays a crucial role in SARS-CoV-2 entry (50). Gallinamide A, a covalent inhibitor of several cysteine proteases including human cathepsin L, was found to inhibit SARS-CoV-2 entry in Vero E6 cells (51). Furthermore, Zhao et al. demonstrated that RNAi-mediated knockdown (KD) of cathepsin L in Huh7 cells reduced SARS-CoV-2 pseudovirus entry in a dose-dependent manner, and overexpression of this cathepsin promoted viral entry. When the activity of cathepsin L was blocked by the inhibitor E64d or amantadine in mice expressing hACE2, SARS-CoV-2 pseudovirus infection was significantly reduced (10). Clinically used glycopeptide antibiotic teicoplanin is known to inhibit the proteolytic activity of cathepsin L. When HEK293T cells expressing high levels of human ACE2 (HEK293T-hACE2^high^) were treated with teicoplanin and its homolog dalbavancin, SARS-CoV-2 entry was attenuated. Teicoplanin pre-treatment was also found to be effective against SARS-CoV-2 infection in mice expressing hACE2 (52). Lamers et al. found that SARS-CoV-2 propagated in VeroE6 cells harbors a point mutation (S686G) adjacent to the RRAR multibasic cleavage site (MBCS) at positions 682–685 on the S protein. The MBCS mutations, including S686G, increased cathepsin usage by SARS-CoV-2 for entry into Vero E6 cells, but not into Vero E6-TMPRSS2 cells (53). Using the CRISPR interference system in ACE2-expressing human-induced pluripotent stem cells (ACE2-iPSCs), Hashimoto et al. found that suppression of cathepsins L and B led to a marked decrease in the SARS-CoV-2 gene copy numbers and the number of cells expressing the viral nucleocapsid protein. The authors also found that dual inhibition of cathepsin B and TMPRSS2 robustly decreased SARS-CoV-2 infection in ACE2-iPSCs (9). Although cathepsins L and B have been found to play an important role during SARS-CoV-2 entry in multiple studies, the possibility remains that the other cathepsins may also be involved. When human-induced pluripotent stem cell-derived pneumocyte-like cells were treated with ONO 5334, a cathepsin K inhibitor, the S protein was improperly processed in the endosome, leading to a 72% reduction in infection compared to control (54). Using a computational platform, a recent study found that the S1/S2 region, which is critical for the activation of the S protein, is susceptible to cleavage by cathepsins L, B, K, S, and V (55). Taken together, the current evidence suggests that SARS-CoV-2 majorly relies on cathepsins B, L, and K for S protein processing during its endocytic entry (Fig. 3A).

### IAV

IAV is an enveloped, negative-stranded RNA virus that belongs to the *Orthomyxoviridae* family. IAV initiates its entry process by binding to sialic acid residues attached to the cell surface glycoproteins and glycolipids. Upon internalization by CME or macropinocytosis, the virus is trafficked to the late endosome. The low pH in the late endosome triggers conformational rearrangements in the trimeric surface glycoprotein HA of the viral particle, converting HA to a fusion-competent conformational state that catalyzes fusion of the viral and endosomal membranes. Viral fusion is followed by viral capsid uncoating and the release of the vRNPs into the cytosol (15). A genome-wide RNAi screen identified cathepsins G and W as potential mediators of IAV infection (56), and a follow-up study confirmed the role of cathepsin W in IAV entry (57). Although depletion of cathepsin W in A549 cells (a human alveolar epithelial cell line) did not impact IAV internalization and HA acidification in the late endosome, viral fusion was remarkably blocked, causing an accumulation of the viral particles inside the late endosomes. Ectopic expression of wild-type but not catalytically inactive cathepsin W in the cathepsin W-deficient cells reversed the fusion defect phenotype, suggesting that the proteolytic activity of the enzyme is crucial for IAV fusion (57). However, the mechanism by which cathepsin W facilitates IAV fusion at the late endosome is currently unknown (Fig. 3B). Recently, the pro-viral role of cathepsin W was confirmed in an animal model. Cathepsin W knockout (KO) mice displayed reduced susceptibility to IAV infection and improved survival in comparison to the wild-type mice, further confirming the importance of cathepsin W in IAV infection (58).

### Severe fever with thrombocytopenia syndrome virus (SFTSV)

Severe fever with thrombocytopenia syndrome (SFTS) is an emerging infectious zoonosis identified in 2009 and is characterized by thrombocytopenia, high fever, multiorgan dysfunction, and a high mortality rate in humans. SFTS is caused by SFTSV, which belongs to the *Phenuiviridae* family. SFTSV is an enveloped, negative-stranded virus with three RNA segments: large (L), medium (M), and small (S). Two glycoproteins, Gn and Gc, encoded by the M-segment of the genome, are embedded in the viral envelope (59). These glycoproteins mediate binding of the virus to the host cell receptors like DC-SIGN and its subsequent internalization (59). Neutralization of endosomal acidic pH by ammonium chloride (NH_4_Cl) or bafilomycin A1 (BafA1) was found to block SFTSV infection, indicating pH dependence of the virus for establishing infection. Although pharmacological inhibition of cysteine cathepsins did not impact SFTSV entry, serine protease inhibitor AEBSF [4-(2-aminoethyl)-benzenesulfonylfluoride hydrochloride] significantly reduced SFTSV infection, suggesting that while cathepsins B and L are dispensable, serine proteases are critical for SFTSV entry (60) (Fig. 3C).

### Chikungunya virus (CHIKV)

CHIKV is a positive-sense, single-stranded RNA virus with enveloped icosahedral capsid, and it belongs to the *Togaviridae* family. After binding to its receptors that include glycosaminoglycans, T-cell immunoglobulin and mucin 1, and matrix remodeling-associated protein 8 (61), CHIKV is internalized by either CME (62) or macropinocytosis (63) or clathrin-independent Eps15-dependent pathways (61, 64). Upon reaching the late endosome, the low pH in the endosome triggers CHIKV fusion, mediated by the envelope (E) protein. Izumida et al. examined the involvement of cathepsin B in CHIKV entry in multiple cell lines and found that either pharmacological inhibition of cathepsin B or its depletion by shRNAs robustly attenuated pseudotyped CHIKV entry. This study also found that when pseudotyped CHIKV particles were incubated with recombinant cathepsin B, the E2 protein of CHIKV was cleaved, generating a novel 19 kDa protein (65). Whether CHIKV entry is also dependent on other cathepsins in addition to cathepsin B remains to be investigated (Fig. 3D).

### Borna disease virus (BDV)

BDV is a neurotropic, non-segmented RNA virus of the *Bornaviridae* family, which attacks the central nervous system and causes behavioral abnormalities. BDV infection manifests a broad range of behavioral alterations, which occasionally progress to severe neurological dysfunctions, leading to fatal outcomes (66). BDV surface glycoprotein G is synthesized as a precursor glycoprotein, which is post-translationally cleaved by furin to generate GP1 and GP2. While GP1 mediates virus binding and internalization, GP2 catalyzes viral fusion at the late endosome, leading to the release of the RNP core into the cytoplasm. RNAi screen against BDV infection in an oligodendroglial cell line identified several pro-viral host factors among which cathepsins L and V were found to mediate BDV entry. The involvement of cathepsin L in BDV entry was further verified by pharmacological inhibition of the endosomal protease by Z-Phe-Tyr(tBu)-diazomethylketone. Treatment of the cathepsin L inhibitor significantly reduced BDV infection in cultured cells, indicating the importance of cathepsin L in BDV entry (67) (Fig. 3E).

### Ebola virus (EBOV)

EBOV is a long, filamentous enveloped virus which belongs to the *Filoviridae* family. It contains a negative-sense, single-stranded RNA genome. The GP, a class I fusion protein present on the virus surface, mediates the attachment of the virus to the host cell receptors and thus facilitates viral entry. EBOV has been reported to bind to several cell surface receptors including C-type lectins, integrins, DC-SIGN, T-cell immunoglobulin and mucin domain 1, and the receptor tyrosine kinase AXL. The surface glycoprotein, GP, is a heterodimer composed of a receptor-binding GP1 subunit and a fusion-mediator GP2 subunit, which remain associated through a disulfide bond (68). After attaching to the cell surface receptor, EBOV majorly uses macropinocytosis to enter the cell. EBOV has also been reported to use CME, although to a limited extent, for cell entry (69). Post internalization, EBOV is trafficked to the acidic late endosome, where cathepsins B and L cleave the GP1 subunit from its initial 130 kDa form to an intermediate 19 kDa fusogenic form, exposing the domain for receptor binding (70, 71). The processed GP1 subsequently binds to Niemann-Pick C1 in the late endosome/lysosome, which leads to GP2-mediated viral fusion at the endosome (72). The requirement of endosomal cathepsins in EBOV entry was demonstrated by Chandran et al. who used vesicular stomatitis virus pseudotyped with EBOV GP (VSV-GP) to investigate EBOV entry in Vero cells (73). They found that treatment of Vero cells with the cysteine protease inhibitor E64d almost completely inhibited VSV-GP infection, suggesting an essential role of cathepsins in EBOV entry. A similar extent of infection inhibition with E64d was observed when more infectious VSV particles were used, which contained a form of GP that lacked the mucin-like/variable (Muc) domain (VSV-GP∆Muc). Furthermore, they blocked the activity of cathepsins B and B/L by CA074 and FYdmk, respectively, using a range of concentrations, and found that the extent of inactivation of cathepsin B, but not cathepsin L, closely correlated with VSV-GP∆Muc infection percentage. In mouse embryonic fibroblasts lacking cathepsin B alone or lacking both cathepsins B and L, VSV-GP∆Muc infection was reduced by >90% and >99%, respectively, indicating an essential role for cathepsin B and an accessory role for cathepsin L in EBOV entry. When purified enzymes were incubated with VSV-GP∆Muc, both cathepsins B and L generated an 18 kDa N-terminal fragment from GP1 (GP1_18K_), but the cleavage efficiency was much higher with cathepsin L than B, which suggests that GP1 cleavage to GP1_18K_ is not the entry step of EBOV that specifically requires cathepsin B. Based on the above observations, the authors hypothesized that the VSV particle with a processed GP1, i.e., GP1_18K_, represents an intermediate form in the cathepsin B-dependent entry pathway. While GP1 cleavage is critical for EBOV entry, no GP1_18K_-dependent enhancement of infection was observed in cells with almost complete activity of cathepsin B or L, indicating that GP1 cleavage is mediated by these cathepsins. The authors proposed that GP1 processing during EBOV entry is a multistep process initiated by cathepsin B and/or L to remove the C-terminal sequences and to generate the N-terminal GP1_18K_, which is further processed by cathepsin B to trigger viral fusion (73). In a subsequent study, Schronberg et al. proposed a two-step model in which cathepsins B and L prime GP1 to generate a 19 kDa intermediate that is acted upon by an endo/lysosomal enzyme to trigger viral fusion (74). The receptor-binding efficiency of the EBOV particles pre-catalyzed with cathepsin L was enhanced, suggesting that cathepsin L-mediated cleavage is important for tight interaction between the virus and its receptor (75). GP mutants P146A/C147A, F153A/H154A, F159A, and F160A showed defective cleavage by cathepsins along with the reduced binding to the host cells (76). Integrin KD/KO resulted in the absence of double chain forms of cathepsins B and L, along with the decreased activity of cathepsin L. Furthermore, pre-primed viruses successfully entered cells in integrin KO/KD conditions, suggesting integrin deficiency has a role in pre-priming the viruses related to cathepsins B and L activity (77). Entry of multiple strains of EBOV was inhibited upon treatment with cathepsins B and L inhibitors in HEK293T and macrophage cells. Sequence analysis of different EBOV strains showed that dependence of viruses on cathepsin B is conferred to by the residue 47 in GP1 and the residue 584 in GP2 (78). GNPTAB is responsible for mannose 6-phosphorylation of cathepsins, and GNPTAB KO cells showed impaired EBOV infection, highlighting the importance of mannose 6-phosphorylation of cathepsins in EBOV infection (79). EBOV passaging in the presence of cathepsins B and L inhibitors (MDL28170, CA-074, CatL inhibitor III) resulted in the accumulation of cathepsin-resistant mutations. Sequencing data showed accumulation of the V37A and S195R mutations in the GP protein. The V37A mutation accumulated in GP upon passaging the viruses in the presence of cathepsins B and L inhibitors, suggesting that single substitution is enough to offer resistance against GP cleavage by cathepsin B/L (80). The A82V mutation in GP of the EBOV Makona strain showed enhanced kinetics and faster fusion, which correlated with efficient processing of GP by cathepsin L (81). Taken together, the evidence suggests the critical roles of cathepsins B and L in the multistep processing of GP1 during EBOV entry (Fig. 3F).

### Human immunodeficiency virus type 1 (HIV-1)

HIV-1 is a retrovirus with two copies of positive-sense, single-stranded RNA genome. HIV-1 has two glycoproteins on the surface, gp120 and gp41, which are embedded in the host-derived lipid envelope. Entry of HIV-1 in the host cell is initiated by binding of gp120 with the surface-expressed CD4, which in turn facilitates interaction with the co-receptors CXCR4 and CCR5. Upon binding to the receptor and co-receptors, the virus fuses at the plasma membrane. Alternatively, the virus can enter the target cell by CME and fuse at the acidic endosome for genome release (82). It was proposed that the interaction of the V3 loop region of gp120 envelope protein with cell surface-associated or endosomal proteases is crucial for HIV-1 entry into the target cells. Schulz et al. investigated the effect of mutations in the gp120 V3 loop region on the susceptibility to proteolytic cleavage by thrombin and cathepsin E and examined the effect of these mutations on HIV-1 infection (83). However, no conclusive evidence was found on the role of cathepsin E in HIV-1 entry. In a similar line, Avril et al. examined the interaction of the cell surface-associated cathepsin G with the gp120 V3 loop, but could not detect any significant cleavage of the V3 loop by the protease in U-937 cells (84). In an interesting observation, El Messaoudi et al. found that incubating HIV-1 with breast milk in lymphocyte culture increased viral growth. The addition of anti-cathepsin D antibody or the cathepsin D inhibitor pepstatin A reversed the enhancing effect of breast milk on viral growth. The authors proposed that cathepsin D may induce conformational changes in gp120, and thus may facilitate the interaction with the co-receptors to facilitate HIV-1 fusion at the plasma membrane (85). Another study investigated the role of cathepsin B in HIV-1 entry and found that specific inhibition of cathepsin B activity by CA-074Me enhances the susceptibility to a CD4-independent HIV-1 strain (mNDK). Conversely, high cathepsin B activity in the endosome was found to reversely correlate with the susceptibility to the mNDK strain, suggesting that in CD4-independent HIV-1 infection, cathepsin B in the endosome functions as a host defense factor (86). Whether cathepsin D is essential for processing gp120, and thereby plays any major role in HIV-1 fusion, remains to be investigated (Fig. 3G).

## ROLE OF CATHEPSINS IN NON-ENVELOPED RNA VIRUS ENTRY

### ReV

ReV is a non-enveloped virus with segmented, double-stranded RNA genome, enclosed in two concentric protein shells. Although ReVs are rarely associated with diseases, they infect a wide range of mammals, including humans (17). Inhibition of cysteine proteases by E64 or a block in endosomal acidification by NH_4_Cl, BafA1, or concanamycin A prevents conversion of ReV to ISVP, suggesting that the σ3 and µ1 cleavage during virion to ISVP conversion is mediated by cysteine proteases in an acid-dependent manner (87). Baer and Dermody observed that although wild-type ReV was susceptible to inhibition by lysosomotropic agents such as NH_4_Cl and cysteine protease inhibitor E64, ReVs with mutations in the outer capsid protein σ3 were resistant to these agents, suggesting that the σ3 mutations selected during persistent ReV infection altered its susceptibility to protease-mediated cleavage during ReV disassembly. The effect of E64 and ammonium chloride disappeared when drugs were added post 60 min after adsorption (88). Sequence analysis of the wild-type and E64-adapted strain showed a common tyrosine to histidine mutation at the 354 position in the S4 gene, suggesting this mutation alters σ3 susceptibility against cellular proteases (89). Other studies also found that cathepsins B, L, and S are important for the disassembly of ReV outer capsid and membrane penetration. Inhibition of cathepsin L activity using inhibitors resulted in decreased viral infection, underscoring the role of cathepsin L in ReV coat disassembly. Cathepsin B and, more efficiently, cathepsin L mediate the conversion of virions to ISVPs (90). Interestingly, although the broad-spectrum cysteine protease inhibitor E64 blocked infection with the ReV serotype 1 Lang in the macrophage-like cell line P388D, treatment with NH_4_Cl did not show any effect. Similarly, cathepsin S inhibitor *N*-morpholinurea-leucine-homophenylalanine-vinylsulfone-phenyl (LHVS) blocked ReV Lang strain infection in the P388D cells in an acid-independent manner. The above results suggest acid-independent but cathepsin B-, L-, and S-dependent processing of the ReV Lang serotype in the P388D cells. Therefore, the processing of ReV outer capsid by cathepsins B, L, and S (Fig. 4A) can be acid-dependent or -independent, depending on the cell line characteristics and viral strains. Furthermore, the authors reported that different strains of ReV undergo cathepsin S-mediated cleavage to different extents, resulting in different levels of infection upon cathepsin S expression (91). Increased survival of cathepsin B^−/−^ mice along with decreased survival of cathepsin L^−/−^ and cathepsin S^−/−^ when infected with the wild-type and ReV T3SA^+^ strain showed the differential influence of cathepsins B, L, and S on the progression of ReV infection (92).

### RV

RV is a non-enveloped, double-stranded RNA virus, which belongs to the *Reoviridae* family. RV is the most common cause of gastroenteritis among infants and young children. There are nine species of RV, of which the A species of RV is responsible for more than 90% infections in humans. The RV particle is composed of six VPs. Among the VPs, the glycoprotein VP7 forms the outer surface and VP4 protrudes from the virion as a spike protein that serves as the attachment protein (18). RVs were earlier proposed to directly penetrate at the plasma membrane, but recent studies have found that depending on the viral strains the viruses can enter via different endocytic pathways (93). After internalization, RV is trafficked to the maturing endosome, transiting through the early endosome. Interestingly, after reaching the maturing endosome, different RV strains follow different trafficking routes to penetrate. The RV strains RRV and SA11-4S exit the maturing endosome in a Rab7-independent manner, whereas the other strains, including nar3, are dependent on Rab7 and reach the late endosome to enter the cytosol (18). A study by Díaz-Salinas et al. revealed that cathepsins are critical for most RV strains to establish infection. Pharmacological inhibition of cathepsins B and L markedly reduced infection with the RV BRV UK strain, but did not display any effect on infection with the RRV or Nar3 strain. Similar RV strain-specific results were obtained by RNAi-mediated depletion of cathepsins B, L, and S. The authors speculated that cathepsins facilitate RV entry by processing the virus surface proteins, thereby promoting the release of the transcriptionally active double-layered particle into the cytosol (94). Similar results were observed for the RV strains Wa, WI61, DA-1, and YM, highlighting the general requirement of cathepsins for RV infectivity (95). However, further investigations will be required to gain deeper insights into the mechanisms of cathepsin-mediated RV infection processes (Fig. 4B).

### Hepatitis E virus (HEV)

HEV is a positive-sense, single-stranded RNA virus that belongs to the *Hepeviridae* family and is a major cause of hepatitis. The single-stranded RNA genome consists of three open reading frames (ORFs): ORF1 (encodes the non-structural polyprotein needed for viral replication), ORF2 (encodes the capsid protein), and ORF3 (encodes a small protein essential for virus secretion). HEV exists as either a quasi-enveloped virus (eHEV) or a non-enveloped/naked virus (neHEV). The host cell entry of HEV initiates with the attachment of the virus to cellular receptors that are not fully characterized. Both eHEV and neHEV enter the host cell via endocytosis with differential kinetics, and after reaching the endosomes, they release their genome into the cytosol (96, 97).

The effect of endosomal pH on HEV genome release remains elusive. Holla et al*.* showed that the HEV uncoating is independent of low endosomal pH (98). In contrast, Fu et al., Yin et al., and Klöhn et al*.* reported the dependence of neHEV infection on low endosomal pH (99–101). Recently, Klöhn et al*.* found the involvement of cathepsin L in HEV infection. KD and KO of cathepsin L showed reduced neHEV infection. Leupeptin, a combined serine and cysteine protease inhibitor, showed significant inhibition of HEV infection. Infection with neHEV was significantly blocked with the treatment of pan-cathepsin inhibitor K11777, cathepsin L inhibitor CAA0225, and cathepsins B and L inhibitor E64d. RNA fluorescence *in situ* hybridization assay of neHEV showed that K11777 and CAA0225 inhibit the dissociation of viral RNA separation from capsid, suggesting the requirement of cathepsins in viral entry. *In vitro* cleavage assay showed that cathepsin L cleaves glycosylated ORF2 into smaller fragments (101). Collectively, these findings suggest an involvement of cathepsin L in HEV capsid processing (Fig. 4C). However, more mechanistic studies are warranted to further examine the requirements of cathepsins in HEV entry.

The roles of cathepsins in the entry of the above viruses are summarized in Table 1.

**TABLE 1:** Details of cathepsin inhibitors with activity in cellular entry of enveloped and non-enveloped RNA viruses

	Virus	Inhibitors	Target cathepsin	Inhibitor concentration	Cell line/model organism	Proposed functions of cathepsin	Ref.
Enveloped virus	SARS-CoV-2	E64d	Cathepsins L, H, B, and calpain	30 μM	HEK293/hACE2	Processing of spike protein	(50)
				20 μM, 30 μM	Huh7		(10)
				12.5 mg/kg body weight	Human ACE2 transgenic mice		(10)
		SID 26681509	Cathepsin L	2 μM	HEK293/hACE2	Activating spike protein for viral and endosomal membrane fusion	(50)
				20 μM	Huh7		(10)
		Gallinamide A	Cathepsin L	10 μM	Vero E6		(51)
		Amantadine	Cathepsin L	300 μM	Huh7		(10)
				50 mg/kg body weight	Human ACE2 transgenic mice		
		Teicoplanin	Cathepsin L	25 μM, 50 μM	HEK293T-hACE2^high^		(52)
				100 mg/kg body weight	hACE2 mice		
		Dalbavancin	Cathepsin L	25 μM, 50 μM	HEK293T-hACE2^high^		
		CA-074	Cathepsin B	30 μM	HEK293/hACE2	Cleavage of spike protein	(50)
		ONO 5334	Cathepsin K	2.5 μM	Vero E6	Processing of spike protein	(54)
	IAV	NA	Cathepsin W	NA	A549	Mediates virus and endosomal membrane fusion	(57, 58)
					C57BL/6 mice		(58)
	SFTSV	NH_4_Cl	Lysosomotropic agent	NA	U373	Reduced infection of SFTSV upon inhibition of serine protease	(60)
		Bafilomycin	Endosomal V-ATPase	NA	U373		
		AEBSF	Serine protease	200 μM	Vero E6		
	CHIKV	CA074Me	Cathepsin B	20 μM, 40 μM	Human 293T, TE671	Digestion of E protein for efficient membrane fusion	(65)
	BDV	Z-Phe-Tyr(tBu)-diazomethylketone	Cathepsin L	4 mg/mL, 6 mg/mL	O1	Processing of BDV surface protein	(67)
	EBOV	Fydmk	Cathepsins B and L	10 μM	Vero	Cathepsin L—plays an accessory role for cleavage of GP1, cathepsin B-initiation of multistep processing of GP, membrane fusion, and uncoating	(73, 78)
		E64d	Cathepsins L and B	300 μM	CHO		(78)
					Vero		(73, 78)
		MDL28170	Calpain, cathepsins B and L	20 μM	Vero E6		(80)
		CatL inhibitor III	Cathepsins B and L	20 μM	Vero E6		(80)
		CA-074	Cathepsin B	80 μM	Vero		(73, 78)
				80 μM	Vero E6		(80)
	HIV	Pepstatin A	Cathepsin D	20	MCF7	Promotes interaction with co-receptors mediated by a conformational change in gp120	(85)
		CA-074Me	Cathepsin B	125 μM	HeLa		(86)
Non-enveloped virus	ReV	NH_4_Cl	Lysosomotropic agent	20 mM	Murine L929 (L)	Disassembly of ReV capsid and membrane penetration	(88)
		E64	Cysteine protease (cathepsin L)	100 μM			(88, 90)
				300 μM	P388D		(91)
		LHVS	Cathepsin S	5 nM			
		CA-074	Cathepsin B	5 μM			
		CA-074Me	Cathepsin B	1 μM	Murine L929 (L)		(90)
		Z-Phe-Tyr(tBu)-diazomethylketone	Cathepsin L	10 μM			
	RV	Leupeptin	Serine and cysteine protease	25 μM	MA104	Processing of viral surface proteins to promote the release of DLPs in the cytoplasm	(94)
		CA-074	Cathepsin B	5 μM	MA104		
		Z-FF-FMK	Cathepsin L	5 μM	MA104		
	HEV	Leupeptin	Serine and cysteine protease	10 μM	HepG2/C3A	Cleavage of ORF2 and release of viral RNA from capsid	(101)

## CONCLUSIONS

Host cell entry represents a key step in the life cycle of viruses. As viruses lack motile or metabolic activity, they are severely limited in their ability to independently initiate cell entry and thus are critically dependent on their hosts to overcome the entry barriers and access the cell interior. Beyond successful internalization, viruses still need to penetrate cellular membranes to liberate their genome into the cytosol, subvert antiviral response, and evade immune detection. Although cells respond to viral invasions by expressing a wide range of protective proteins, viruses have also evolved strategies to manipulate the host defense responses by tapping into the functions of specific host molecules which, instead of restricting the viruses, offer favorable support during their entry and replication. Among the host molecules that viruses exploit to facilitate their entry are cathepsins, which are proteolytic enzymes of the endo/lysosomal system. In this review, we highlighted the critical roles of cathepsins in the host cell entry of enveloped and non-enveloped human pathogenic RNA viruses. However, our understanding of the functions of cathepsins in the entry of many of the viruses discussed here is far from complete.

The majority of the studies focused only on a few cathepsins in the context of viral entry, leaving the involvement of other potential cathepsins unexplored. Whether the other cathepsins than the ones studied are also exploited by a specific virus for entry is currently unknown. To elucidate the functions of cathepsins in viral entry, most of the studies relied on cathepsin inhibitors, with relatively few studies employing genetic perturbation methods, and even fewer combining both approaches. Since only a few cathepsin inhibitors specific to selected members of the cathepsin family are currently available, the roles of the other cathepsins for which inhibitors are unavailable remain poorly understood. To address these gaps, efforts should be directed at designing molecules that can specifically target and inhibit the activity of each member of the cathepsin family. Furthermore, systematic investigations into the functions of each cathepsin against a particular virus using genetic manipulation tools such as siRNAs, shRNAs, or CRISPR/Cas9 should be carried out for a comprehensive evaluation of all the cathepsins, rather than focusing on a select few. Despite these gaps in our knowledge, recent studies have identified several cathepsins that promote the entry of several pathogenic viruses. Since blocking viral entry effectively inhibits infection at an early stage and since many viruses exploit common pathways for entry, it is conceivable that targeting cathepsins individually or in combination by specific inhibitors can potentially block the entry of a broad range of viruses.
